# Community-based pulmonary rehabilitation for post-TB lung disease – a programmatic intervention

**DOI:** 10.5588/ijtldopen.25.0201

**Published:** 2025-08-13

**Authors:** B. Stenberg, M. Drage, B. Matewere, V. Alvarez-Manon

**Affiliations:** ^1^ LHL International Tuberculosis Foundation, Oslo, Norway;; ^2^ Paradiso TB Patient Trust, Lilongwe, Malawi.

**Keywords:** tuberculosis, Malawi, chronic respiratory disease, post-TB sequalae, PTLD, 6MWT

## Abstract

**BACKGROUND:**

Many survivors of pulmonary TB struggle with poor lung health and poor quality of life.

**METHODS:**

We designed and implemented a culturally appropriate, low-cost, community-based pulmonary rehabilitation (PR) program, and measured its effect on health and wellbeing. We identified former TB patients with pulmonary complaints in 9 districts in Malawi. Those who met the inclusion criteria were divided into groups and given a tailored training program with an educational component and guided through sessions twice per week for 12 weeks. Data on 13 health variables was collected before and after the PR.

**RESULTS:**

467 former TB patients were enrolled. 285 (61%) were female. The mean age was 45 years (16–81). After the 12-week PR, chest pain reduced from 66.4% (310) to 8.8% (41) and cough from 47.5% (222) to 9.6% (45). Moderate and severe dyspnea disappeared, and no one scored below 80 on the Karnofsky Index scale after the PR ended. Endurance and functional capacity measured by 6-minute walk test (6MWT) increased by 15.5%.

**CONCLUSION:**

The 12-week course of PR had a positive effect on people’s health and well-being, and it is now integrated into the National TB strategy in Malawi. The benefits of such programs are highly significant for the individual and the broader community. We strongly encourage other countries to implement similar PR programs.

Follow-up and management of TB survivors has long been ignored, especially in low-income countries (LICs) such as Malawi. The traditional focus in management of TB has been on finding and treating those who are sick – and national and global data on TB is typically based on incidence and treatment outcomes. Thus, follow-up of patients has normally ended with TB treatment completion, leaving millions of survivors in poor health, and with no follow-up.

‘When we started tuberculosis treatment, no-one told us that it would never leave us”^1^

A scoping review published in 2018^2^ concluded that pulmonary TB (PTB) is an important risk factor for chronic respiratory disease due to residual lung damage. However, the article noted that the WHO End TB strategy did not mention post-TB chronic lung disorders and that programmatic interventions to address these were lacking. In recent years, we have seen a dramatic increase in reports on the disability burden of TB^3^ as a direct consequence of the disease or its treatment, and a growing concern and interest in the topic. It is estimated that 47% of TB disability adjusted life years (DALYs) are attributable to post-TB sequalae.^4^ Accordingly, we have seen increased awareness, exemplified by the second international symposium on post TB in 2023^5^ and a clinical statement on ‘Post-TB health and wellbeing’.^6.^ In addition, WHO has launched its first ever policy brief on TB-associated disability,^7^ and Brazil^8^ and the Latin American region^9^ recently published guidelines for the management of post-TB lung disease (PTLD).

A metanalysis reported mental health disorders as the most common type of disability (23,1%) followed by respiratory impairment (20,7%) and musculoskeletal impairment (17,1%).^10^ The prevalence of disability is inversely related to the country’s income level, probably due to the high burden of TB in LICs, diagnostic delays and barriers in accessing health care, and/or low health service coverage. This is why it is crucial that strategies to address TB related disability can also be implemented at primary health care and community level. Specifically, PTLD, is defined as the presence of respiratory abnormalities, with or without symptoms, partly due to the history of TB. A multicenter study^11^ found that a history of TB increased the risk for obstructive airway disease by 2.5-fold, independent of smoking and other risk factors. Other studies investigated the clinical patterns in PTLD.^12^ A study in Malawi^13^ found that 60.7% of the cohort had respiratory symptoms after successful treatment of PTB. This decreased to 30.7% a year after TB treatment completion. Some studies also suggest women bear a disproportionally higher burden of disability and report lower improvements in health-related quality of life (HRQoL).^14^

Available data supports the use of pulmonary rehabilitation (PR) in a variety of chronic respiratory conditions,^15,16^ including for PTLD.^17^ The benefits of PR in patients with PTLD were recently demonstrated through a prospective multicenter study,^18^ but programmatic interventions to address this are still lacking. Overall, there is less evidence on rehabilitation and management for TB sequalae in sub-Saharan Africa and other resource limited settings. We aimed to contribute to reducing this gap by documenting and assessing the intervention and its effect on people’s health in Malawi.

## METHODS

**Figure 1.**
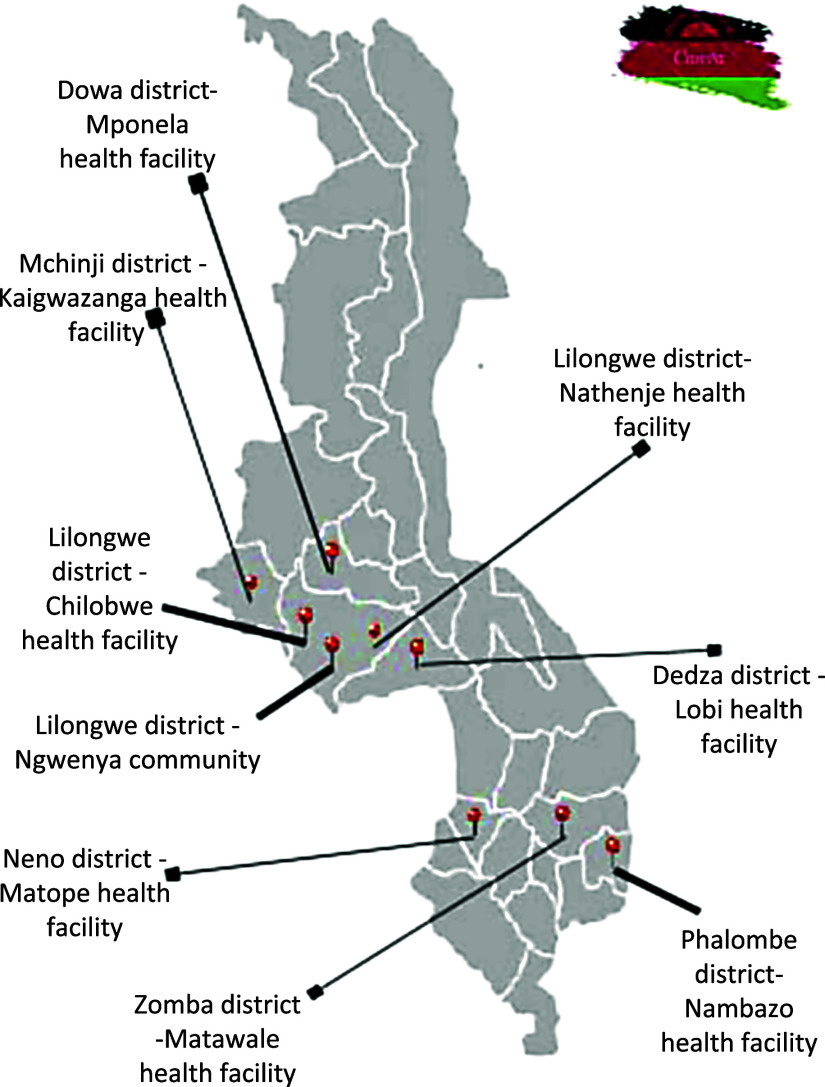
Map showing the nine sites in Malawi where PR was implemented.

A programmatic intervention was implemented between 2020 to 2023 in 9 districts in Malawi (Chilobwe, Dedza, Dowa, Matawale, Mchinji, Nambazo, Nathenje, Neno and Ngwenya; see in map Figure 1). We designed a community-based PR program that would allow us to map health issues faced by former TB patients, and to measure the effects of PR on several physical metrics. At the beginning of, and during, the intervention, there were no protocols or guidelines for follow-up of patients after cure of TB in Malawi, and no PR program existed. The project thus implemented pioneer work aimed at demonstrating by proof of concept the feasibility and effectiveness of a low-cost PR. This was done in agreement with the district and national health authorities, and with the collaboration of local physiotherapists, Paradiso TB Patient Trust, the National Organization of Nurses and Midwives (NONM) and staff from the local health clinics.

### PR program

Thorough training of all facilitators and managers of the PR was conducted, and a total of 467 former TB patients with lung complaints were enrolled in the program. Directly observed exercise sessions were done in groups of 16–25 people, in an outdoor open space close to the local clinic, twice a week for 12 weeks. It focused on circular training with various exercises to help increase both the endurance and strength of participants. Mainly locally made equipment was used to facilitate the sustainability of the PR. All participants received funds to cover transport to and from sessions. The therapy was individually tailored to each participant based on collected baseline data concerning their health status. Each session also included a short health education component, done in groups. Data was collected before and after the PR, using a structured questionnaire in the local language (Chichewa) with three categories: self-reported symptoms, clinical signs, and measures of functional capacity. We did not perform pulmonary function tests (PFTs) such as spirometry due to the lack of equipment at local level. In total, 13 health related variables were collected before the start of PR and again after 12 weeks of PR and analyzed using SPSS (Version 29.0.20). Because the PR was designed with repeated measures, a paired sample t-test was conducted to evaluate whether a statistically significant difference exists between the mean variables measuring health status before and after 12 weeks of community-based pulmonary rehabilitation. Frequency tests and cross tab analysis were also done.

### Ethical statement

The project received approval from the National Tuberculosis and Leprosy Elimination Programme (NTLEP). Oral informed consent was obtained from all participants prior to the collection and use of their health data. As this was a programmatic project and not a research study, in accordance with local regulations, institutional ethics committee approval was not required.

## RESULTS

**Figure 2.**
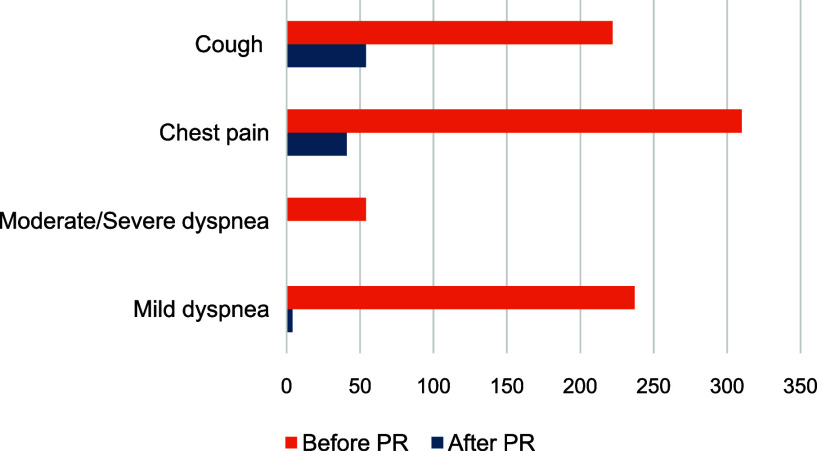
Number of people experiencing symptoms before and after 12 weeks of pulmonary rehabilitation (PR).

The data set includes responses from a total of 467 former TB patients participating in PR at nine different health centers. 61% (285) were female and 39% (182) were male. The median age was 45 (16–81). Before the start of the PR, chest pain 66,4% (310/467) and cough 47,5% (222/467) were the most common complaints. Both symptoms decreased substantially after the 12 week (12w) program to 8,8% (41/467) and 9,6% (45/467) respectively, see Figure 2. There were no significant differences between male and female or age groups. However, there were greater improvements in symptoms among the elderly (> 60).

“My body ached the first day, but I got better and stronger. I also felt happier.” (participant of PR)

Dyspnea (difficulty breathing) was measured using the modified Medical Research Council (mMRC)^19^ score. Analysis showed that before enrolling in the PR, 11.5% (53/467) and 50.7% (237/467) of participants had moderate/severe or slight dyspnea respectively. After the intervention this dropped drastically to 0% and 0.9% (4/467) – see Figure 2. We also looked at functional capacity or impairment.^20^ Before PR, 27% (126/467) scored between 50–70 on the Karnofsky scale, with the highest scores among the highest age group with 44.6% (25/56). A score between 50–70 means a person is unable to work or carry out normal activities but is still able to live at home and care for most of their personal needs. After 12 weeks of PR all participants scored between 80–100 on the scale, showing an impressive improvement in functional capacity.

**Figure 3.**
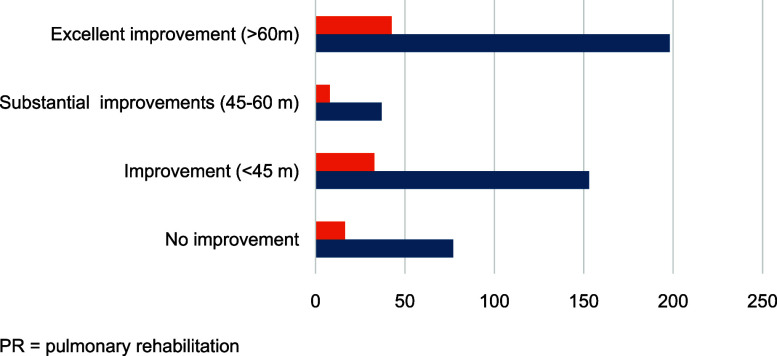
6-minute walk test comparing pre-PR (orange) to post PR (blue). The line below shows number of people in each group. PR = pulmonary rehabilitation

To further assess endurance and functional capacity, a 6-minute walk test (MWT)^21^ was also done before and after the 12 weeks program of PR. The mean distance walked at baseline (400 m) increased by 15.5% at endline (462 m). The difference in meters between pre-PR and post-PR was calculated for each participant, and a recoding done into four groups: No improvement or decline, 16.6% (N 77); improvement up to 45m, 2.9% (N 153); improvement from 45–60m, 8.0% (N 37); and improvements above 60 m, 42.6% (N 198). An improvement above 45 m is considered good and indicates lasting effect.^22^ Our study reveals that half of the participants (50,6%) showed a significant improvement, and as many as 83.4% walked more meters after the rehabilitation, compared with before – see Figure 3.

Overall, the analysis clearly shows a difference between pre- and post-PR on symptoms and activity level, suggesting improvements in participants’ health after 12 weeks of PR in all areas of health measured. Key variables such as chest pain, cough, dyspnea and activity level measurements all suggest positive results, which go beyond people’s health and have a positive impact on their daily lives and general well-being.

Analysis show that participants’ vital signs, strength, endurance and functional capacity was significantly improved after PR. In 10 out of 13 health variables the changes reported showed statistically significant improvements (with 9 variables with p-value < 0.001 and one variable with p value of 0.002). Only the smaller positive changes seen in respiratory rate (RR), oxygen saturation (O2Sats) and hemoptysis were not statistically significant (the latter with p> 0.059). The Table shows the results of a paired sample t-test and the P value (significance) for all health parameters measured before and after PR.

**Table.** Results of paired sample t-test demonstrating significance before and after pulmonary rehabilitation.

Paired samples t test	Mean (M)	Standard deviation (SD)	t-value (t)	Degree of freedom (df)	Significance (P-value)
RR	−0,33	4,213	−1,691	466	0,091 (n.s)
PR	1,835	12,935	3,066	466	0,002**
O2Sats	−0,191	3,162	−1,303	466	0,193 (n.s.)
BMI	−0,2007	1,16587	−3,72	466	<,001***
Dyspnea	0,735	0,653	24,346	466	<,001***
Chest pain	0,576	0,52	23,934	466	<,001***
Chest pain score	2,218	2,083	23,016	466	<,001***
Cough	0,379	0,571	14,345	466	<,001***
Hemoptysis	0,017	0,196	1,891	464	0,059 (n.s.)
RPE	1,63	4,081	8,629	466	<,001***
6MWT	−61,7154	90,8711	−14,677	466	<,001***
1 ARM	−1,458	1,068	−29,495	466	<,001***
Karnofsky scale score	−18,415	9,96	−39,956	466	<,001***

* p<0,05,

** p<0,01,

*** p<0,001, n.s = not significant.

RR = respiratory rate; PR = pulse rate; O2Sats = oxygen saturation; BMI = body mass Index; RPE = rate of perceived exertion; 6MWT = 6-minute walk test; 1 ARM = maximum lifting of weight with one arm.

## DISCUSSION

Data shows that after cure, TB pivots from being the deadliest infectious disease, to being a significant contributor to the global burden of non-communicable diseases, especially chronic lung disease.^23^ Reducing the burden of TB sequalae is therefore important, both by preventative measures such as addressing known risk factors (like diagnostic delays and repeated episodes of TB), and by ensuring post TB care by offering continued support for TB survivors, such as the low-cost community lead PR.

As has been shown above, the improvements within each of the metrics is remarkable, and preliminary data from a nested thematic analysis on the impact in quality of life, suggests that the effect of PR not only prevails, but goes beyond the physical benefits, with a positive psychological and social impact. Classical symptoms such as chest pain, cough and dyspnea greatly improved. Strength and endurance, measured by 6MWT and 1 RM, also improved significantly. One participant put it this way:

“After being cured of TB, I never thought anyone would think of helping me with the problems I was facing post TB. I could not sleep on one side, but after the exercises (PR) I am able to.”

## CONCLUSION

TB affects not just the individual, but their households and communities too, often for years. The 12-week PR program in Malawi, had a significant positive effect on people’s health and well-being, contributing to the increasing evidence on the importance of continued support to patients after TB treatment. PR therefore benefits not just the individual, but also the broader community. We encourage all National TB programs to include PR programs in their TB control strategies, and for researchers to investigate the potential benefits of initiating PR during TB treatment.
